# Matched-Pair Analysis: Large-Sized Defects in Surgery of Lower Limb Nonunions

**DOI:** 10.3390/jcm12134239

**Published:** 2023-06-23

**Authors:** Sebastian Findeisen, Melanie Schwilk, Patrick Haubruck, Thomas Ferbert, Lars Helbig, Matthias Miska, Gerhard Schmidmaier, Michael Christopher Tanner

**Affiliations:** University Hospital Heidelberg, Clinic for Trauma- and Reconstructive Surgery, Center for Orthopaedics, Trauma Surgery and Paraplegiology, Schlierbacher Landstraße 200a, 69118 Heidelberg, Germany; sebastian.findeisen@med.uni-heidelberg.de (S.F.); melaniesonja.schwilk@med.uni-heidelberg.de (M.S.); patrick.haubruck@sydney.edu.au (P.H.); thomas.ferbert@med-uni-heidelberg.de (T.F.); lars.helbig@med.uni-heidelberg.de (L.H.); matthias.miska@med.uni-heidelberg.de (M.M.); gerhard.schmidmaier@med.uni-heidelberg.de (G.S.)

**Keywords:** diamond concept, Masquelet technique, nonunion, nonunion therapy, segmental bone defect

## Abstract

Background: The treatment of large-sized bone defects remains a major challenge in trauma and orthopaedic surgery. Although there are many treatment options, there is still no clear guidance on surgical management, and the influence of defect size on radiological and clinical outcome remains unclear due to the small number of affected patients. The aim of the present study was to determine the influence of defect size on the outcome of atrophic and infected nonunions of the tibia or the femur based on the diamond concept in order to provide recommendations for treatment guidance. Patients and Methods: All medical records, surgical reports, laboratory data and radiological images of patients treated surgically for atrophic or infected nonunions of the lower limbs (femur or tibia) between 1 January 2010 and 31 December 2020 were examined. Patients with proximal, diaphyseal or distal nonunions of the femur or tibia who were surgically treated at our institution according to the “diamond concept” and attended our standardised follow-up program were included in a database. Surgical treatment was performed as a one- or two-step procedure, depending on the type of nonunion. Patients with a segmental bone defect ≥5 cm were matched with patients suffering a bone defect <5 cm based on five established criteria. According to our inclusion and exclusion criteria, 70 patients with a bone defect ≥5 cm were suitable for analysis. Two groups were formed by matching: the study group (bone defect ≥5 cm; n = 39) and control group (bone defect <5 cm; n = 39). The study was approved by the local ethics committee (S-262/2017). Results: The mean defect size was 7.13 cm in the study and 2.09 cm in the control group. The chi-square test showed equal consolidation rates between the groups (SG: 53.8%; CG: 66.7%). However, the Kaplan–Meier curve and log-rank test showed a significant difference regarding the mean duration until consolidation was achieved, with an average of 15.95 months in the study and 9.24 months in the control group (α = 0.05, *p* = 0.001). Linear regression showed a significant increase in consolidation duration with increasing defect size (R^2^ = 0.121, *p* = 0.021). Logistic regression modelling showed a significant negative correlation between consolidation rate and revision performance, as well as an increasing number of revisions, prior surgeries and total number of surgeries performed on the limb. Clinical outcomes showed equal full weight bearing of the lower extremity after 5.54 months in the study vs. 4.86 months in the control group (*p* = 0.267). Conclusion: Surprisingly, defect size does not seem to have a significant effect on the consolidation rate and should not be seen as a risk factor. However, for the treatment of large-sized nonunions, the follow-up period should be prolonged up to 24 months, due to the extended time until consolidation will be achieved. This period should also pass before a premature revision with new bone augmentation is performed. In addition, it should be kept in mind that as the number of previous surgeries and revisions increases, the prospects for consolidation decrease and a change in therapeutic approach may be required.

## 1. Introduction

Fractures of long bones are a common injury in all age groups, and it is not unusual for their treatment to be complex and associated with various complications such as delayed union, nonunion or deep infection [1]. Although there is still no uniform definition of nonunion, the current definition of the European Society of Tissue Regeneration in Orthopaedics and Traumatology (ESTROT), which defines a nonunion as a fracture that will not heal without further intervention regardless of the duration of treatment, has gained acceptance [2]. The risk of developing a nonunion per fracture is reported to be approximately 2% for the scaphoid, the tibia and the femur [3,4]. Depending on the localisation of the fracture and the individual risk profile, nonunion rates of up to 30% have been reported [4,5]. Due to patient-related risk factors such as sex, age, smoking status, diabetes and vascular status and general risk factors such as fracture localisation, high-energy trauma, open or multifragmentary fractures, deep infections, osteomyelitis and nontraumatic tumour resections, long bone fractures may even result in critical-size bone defects [6,7,8,9]. In general, there are still major differences and discrepancies in the definition of large-sized bone defects. Lindsey et al. declared these as bone defects whose length exceeds 2–2.5 times the diameter of the affected bone [10]. Haines et al., on the other hand, defined extensive or critical bone defects as those that do not heal spontaneously during a patient’s lifetime using standard treatments [11]. Nonunions and especially large-sized nonunions often require a larger number of operations, which have a high financial impact on the health care system, with average treatment costs exceeding $10.000 per nonunion (USD $11.333, CAD $11.800 and GBP £29.204) [12]. Even more important is the impairment of the patient’s quality of life, social participation, socioeconomic level and independence, which are significantly limited by nonunions [12,13,14]. The most common treatment options for critical-size bone defects are autologous cancellous bone grafting [15,16], cortical allografts [17], vascularised bone transfers [18] and distraction osteogenesis (e.g., Ilizarov bone transport method) [19]. In the last few years, the “diamond concept” has become an established treatment modality for nonunions due to high consolidation rates and satisfactory clinical results [14,20,21]. First described by Giannoudis et al. using a one-step or two-step procedure, the diamond concept combines the considerations of mechanical stability, improvement of vascularisation, use of osteoconductive scaffolds and additional growth factors to ensure optimal conditions for fracture healing [22,23]. The “induced membrane” or Masquelet technique as a two-step procedure combines foreign-body-induced membranes and cancellous autografts allowing the reconstruction of large defects or infected nonunions, providing good vascularisation without the risk of rapid graft resorption [24,25]. Due to the small number of patients with segmental bone defects, accurate data on the influence of defect size on consolidation and clinical outcome are lacking, making the reconstruction of segmental bone defects one of the greatest challenges in orthopaedic surgery. Therefore, the following study aims to assess the influence of defect size on the clinical and radiological outcomes of atrophic and hypertrophic nonunions treated according to the diamond concept with a one- or two-step procedure, utilising a matched-pair analysis. For this purpose, the primary objective was defined as consolidation and, in particular, the influence of defect size (cm) on consolidation. The duration to consolidation, the duration to full weight bearing and the influence of general risk factors on consolidation were investigated as secondary objectives.

## 2. Material and Methods

### 2.1. Study Design

The current study was designed as a single-centre retrospective matched-pair analysis based on a clinical database and was conducted in accordance with the Declaration of Helsinki. Ethical approval was granted by the local ethics committee (S-262/2017).

Patients were recruited at the hospitals of the University of Heidelberg in the period from January 2010 to December 2020 with a minimum follow-up of 12 months if consolidation was not achieved before. All patients with proximal, diaphyseal or distal nonunions of the femur or tibia who were surgically treated at our institution according to the “diamond concept” and attended our standardised follow-up program were identified as potentially suitable for analysis in the current study. Patients were matched based on established criteria including sex, age, affected long bone and exact localisation, surgical treatment, smoking status and history of infection. Any medical records, study questionnaires and radiological images of patients treated surgically for atrophic or infected nonunions of the lower limb between January 2010 and December 2020 were examined. Surgical treatment was performed as a one- or two-step procedure, depending on the type of fracture, and strictly followed the recommendations of the “diamond concept”. Therefore, atrophic and hypertrophic nonunions were included. Exclusion criteria were minority, nonunions of other anatomical locations, a follow-up less than 12 months if consolidation had not taken place beforehand, primary joint replacement of the affected anatomical region or compound osteosynthesis as well as primary amputation of the limb. Patient data were carefully reviewed regarding patient characteristics, type of surgical treatment, mechanical stability, presence of infection and radiological sings of consolidation. Patients with a segmental bone defect ≥5 cm were matched with patients suffering a bone defect <5 cm. The defect size was determined by X-ray and CT scan. For this purpose, the bone defect was viewed in all planes and the maximum length of the cortical interruption of the nonunion was measured.

**Figure 1:**
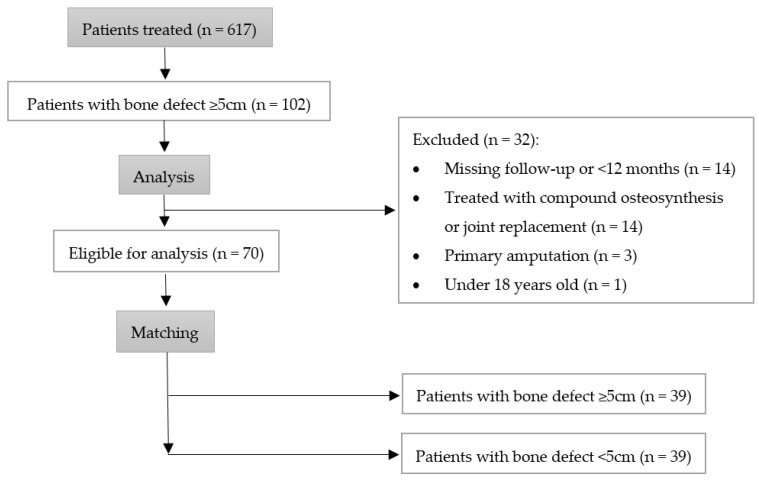
Flow chart visualising the patient selection process.

Between 1 January 2010 and 31 December 2020, 617 patients with a nonunion of the femur or the tibia underwent surgery at our institution. On reviewing the radiological findings, we found 102 patients with segmental bone defects larger than 5 cm. According to the exclusion criteria, 32 patients had to be excluded from the current study (14 due to a loss to follow-up, 14 patients were primarily treated with a joint replacement or compound osteosynthesis, 3 patients underwent a primary amputation of the limb, and 1 patient was under 18 years old), resulting in 70 suitable patients. The eligible patients were then matched with patients suffering a nonunion with a bone defect <5 cm based on the matching criteria. We were able to find suitable partners for 39 patients of the study group, resulting in a total of 78 patients included in this study (Figure 1). The preoperative general physical condition of the patients was assessed using the ASA score as an established clinical tool [26]. Further data showing patient characteristics are found in Table 1. 

### 2.2. Matching

Overall, two groups were analysed and compared in the current study. 

First, the study group, including patients who received surgical treatment based on the diamond concept with segmental bone defects ≥5 cm. Second, the control group, whose members were surgically treated based on the diamond concept with a bone defect <5 cm. 

Matching was performed by an experienced clinician blinded towards clinical and radiological results. Patients were matched based on established criteria including sex, age, affected long bone and exact localisation (proximal, diaphyseal, distal), surgical treatment (one-step vs. two-step procedure), smoking status (smoker, previous smoker, nonsmoker) and history of infection (infected, noninfected). If more than one matching partner was found, secondary matching criteria such as the presence of diabetes mellitus, application of BMP2 or BMP7, use of bone graft substitutes (Vitoss^®^, Vitoss-BA, Mahwah, NJ, USA; Bioglass^®^, Bonalive Biomaterials Ltd., Turku, Finland), type of autologous bone graft (RIA, iliac crest, iliac crest + RIA) and type of fracture (closed vs. open fracture) were used to find the most similar matching partner. Of the total 70 suitable patients, 31 were lost due to a missing partner, so two groups with n = 39 patients each could be formed. 

### 2.3. Surgical Technique 

Depending on patient characteristics, type of fracture or rather type of nonunion, presence of infection and defect size, either a one-step or a two-step procedure was performed. 

The one-step procedure was used during the early stage of the diamond concept (in 2010 and 2011) and was later replaced by a two-step-procedure. It consisted of removal of existing osteosynthesis material, radical debridement of the avital bone and surrounding avital soft tissue, tissue sampling for microbiological testing, transplantation of autologous bone graft and optional application of growth factors (rhBMP-2 or rhBMP-7). Biomechanical stability was achieved by de novo osteosynthesis using plates, nails or screws. 

The two-step procedure was performed using the induced membrane or Masquelet technique. This procedure was applied to infected or atrophic nonunions with large-sized defects or if the soft tissue status was poor. The first surgical step also consisted of removal of existing osteosynthesis material and radical debridement down to the healthy bone. Furthermore, tissue samples for microbiological testing were taken. The defect was then filled with polymethyl-methacrylate (PMMA) impregnated with gentamycin and/or vancomycin as appropriate. If infection was detected, the first step was repeated, with the cement spacer changed during each procedure, until asepsis was achieved and confirmed by negative microbiological results. Afterwards, the spacer was left in situ for 6–8 weeks, allowing the resulting foreign body reaction to induce formation of the vascularised Masquelet membrane.

During the second step of Masquelet therapy, the spacer was carefully removed with the best possible protection of the membrane, and once again, debridement of the avital bone and soft tissue was performed. The resulting defect was filled with a combination of autologous bone graft (e.g., from the iliac crest, RIA of the femur or tibia), a bone substitute (Vitoss^®^, Vitoss-BA, Bioglass^®^) and, if necessary, additional growth factors (BMP7 or BMP2). Depending on nonunion characteristics, de novo osteosynthesis was either performed during the first or the second step using plates, nails or screws. 

Regardless of the type of procedure, the collected tissue samples were directly processed following the standard of care in our microbiological department. If obligate pathogen bacteria were detected, or in the case that more than two independent samples were positive for the same facultative pathogen bacteria, evidence of infection was confirmed. After tissue sampling, 1.5 g of cefuroxime was administered preoperatively, and antibiotic therapy was continued three times daily until the results from the microbiology department were available. If infection was detected, antibiotics were adjusted based on the sensitivity of each bacteria detected, and antibiotic therapy was continued for at least several weeks or until serum C-reactive protein (CRP) levels normalised and wound healing was complete. 

If infection was detected after the one-step procedure, antibiotic therapy was performed and the CRP level and wound situation monitored. For persisting infection, revision surgery had to be conducted, performing another one-step procedure or switching to a two-step procedure depending on patient characteristics, infection status and the discretion of the responsible surgeon. Helbig et al. published further information about antibiotic treatment standards in our institution [27]. 

**Figure 2:**
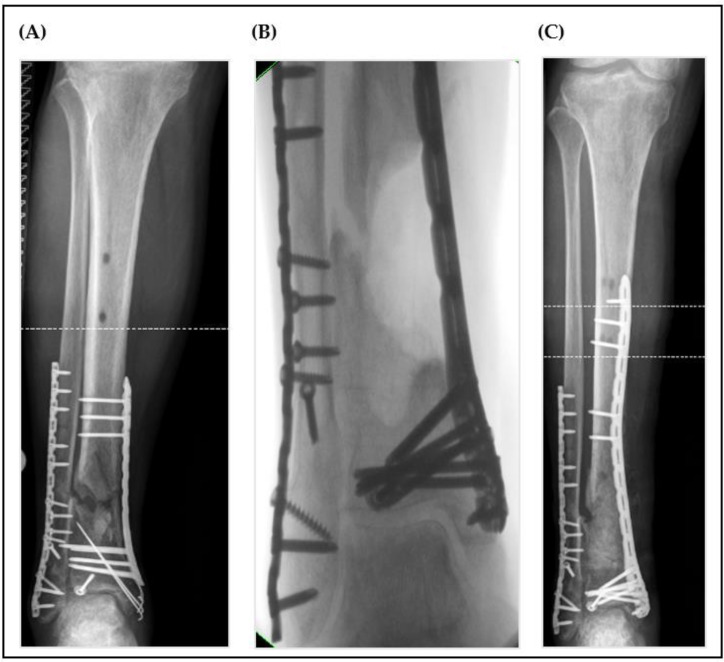
Radiological images of a nonunion of the distal tibia (**A**) with segmental bone defect 6 months after second-degree open tibial fracture due to a motorcycle accident (study group case). The intraoperative findings (**B**) after the radical debridement revealed a large-sized bone defect which was then filled with a PMMA cement spacer and left in situ for six weeks. During Masquelet step 2, the bone defect was augmented using autologous cancellous bone from the femur (RIA) and tricalciumphosphate with 10% bioglass additive (Vitoss-BA^®^). The postoperative radiological control can be seen in (**C**).

**Figure 3:**
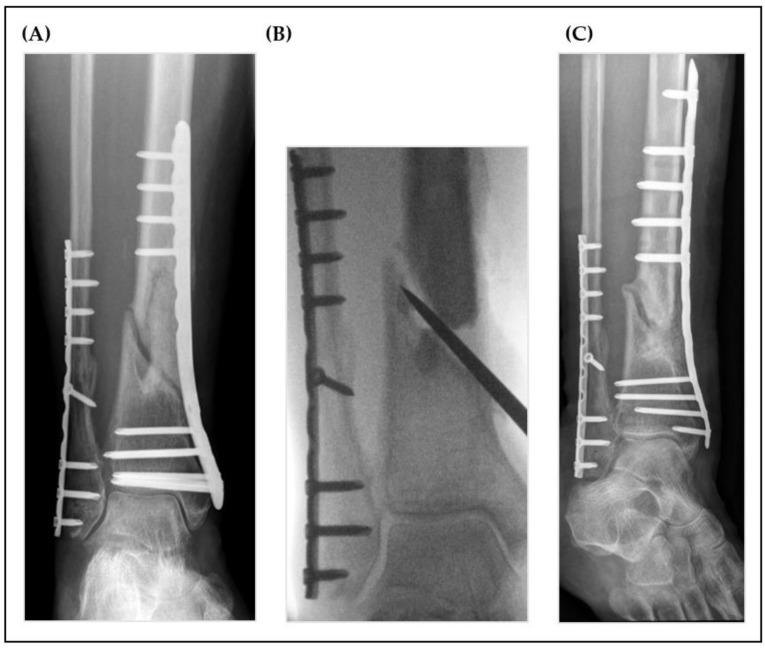
Radiographs of a nonunion of the distal tibia (**A**) 9 months after distal tibial fracture (control group case). The intraoperative findings after radical debridement (**B**) revealed a small bone defect, which was filled with a PMMA cement spacer that was left in situ for 6 weeks. During Masquelet step 2, the bone defect was augmented using autologous cancellous bone from the femur (RIA) and tricalciumphosphate (Vitoss^®^). The postoperative radiological control can be seen in (**C**).

Radiological examples of nonunion treatment for each group are shown below (Figure 2, Figure 3).

### 2.4. Postoperative Care and Determination of Outcome

All patients who underwent surgical nonunion treatment were included in our structured follow-up program. Therefore, clinical and radiological examination was performed 6 weeks, 3, 6 and 12 months and then annually after surgery. Collected information was time until full weight bearing and radiological status (X-ray or CT scan). The clinical and radiological outcome was evaluated 12 months after the final surgical treatment. Radiological criteria included disappearance of former fracture lines, presence of bridging callus, number of bridged cortices and loosening or even failure of the implant. A nonunion was considered consolidated if three of the four cortices were bridged in a conventional X-ray or CT scan. As a clinical criterion, the duration until full weight bearing of the affected extremity was used. If consolidation was reached earlier, a 12-month follow-up did not necessarily have to be obtained. X-rays and CT scans were assessed independently by two different experienced and blinded trauma surgeons. Finally, collected data were entered into a database for further statistical analysis. 

### 2.5. Statistics

For the primary objective, the chi-square test was used to show differences in consolidation rates between the two groups. In addition, linear regression was used to evaluate the influence of increasing defect size on the consolidation. For the secondary objectives, the Kaplan–Meier curve and log-rank test were used to show differences in the mean duration to consolidation and the mean duration to full weight bearing. Furthermore, logistic or linear regression analysis was performed to assess the influence of the individual risk factors on consolidation. For this purpose, a group-specific and a cross-group analysis took place in each case. The parameters examined were defect size, smoking status, infection status, presence of diabetes mellitus, fracture type, number of previous surgeries, number of revisions, number of total surgeries as well as the intake of NSAIDs, steroids or drugs. In addition, logistic regression was used to evaluate differences in surgical procedure on consolidation (one-step vs. two-step procedure, autologous bone graft, bone graft materials, use of BMP2 or BMP7, type of osteosynthesis). Statistical analysis was performed utilising IBM SPSS Statistic Version 29 for Windows by the authors in cooperation with the Statistical Institute of the University of Heidelberg. The level of significance (α) was set at 5%, and continuous variables are shown with the absolute mean ± standard deviation (SD). All *p*-values reported are to be interpreted descriptively as they were not adjusted for multiple testing.

## 3. Results

### 3.1. Radiological and Clinical Nonunion Therapy Outcome

**Figure 4:**
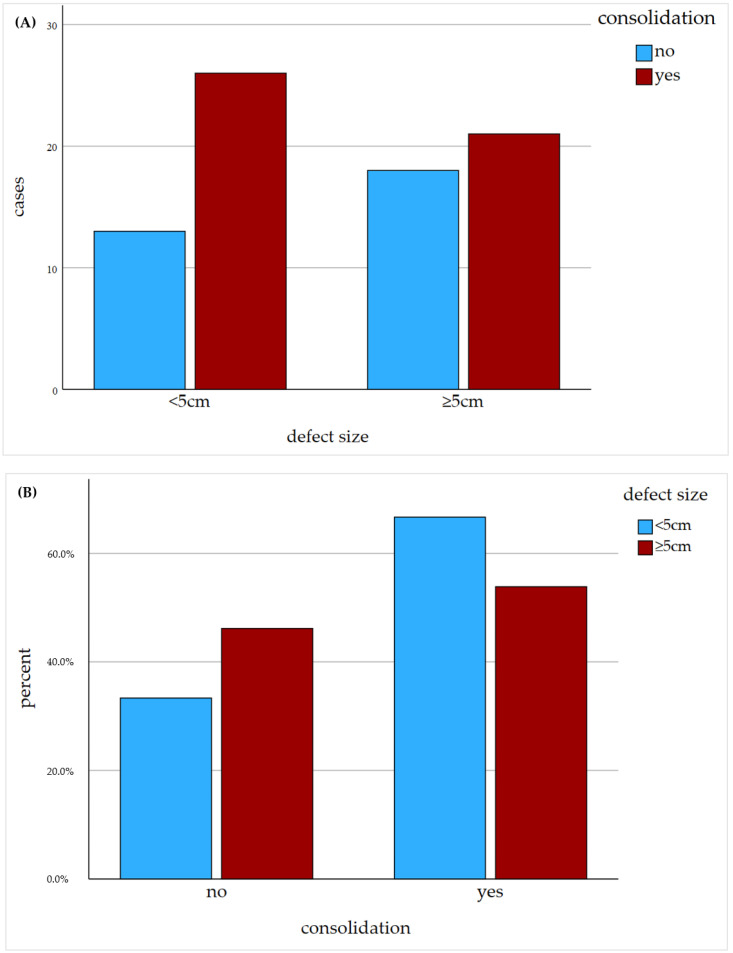
Radiological outcome of nonunion treatment based on the “diamond concept”. The consolidation subsequent to the nonunion therapy in respect to the defect size is shown in absolute numbers (**A**) and percentage (**B**).

**Figure 5:**
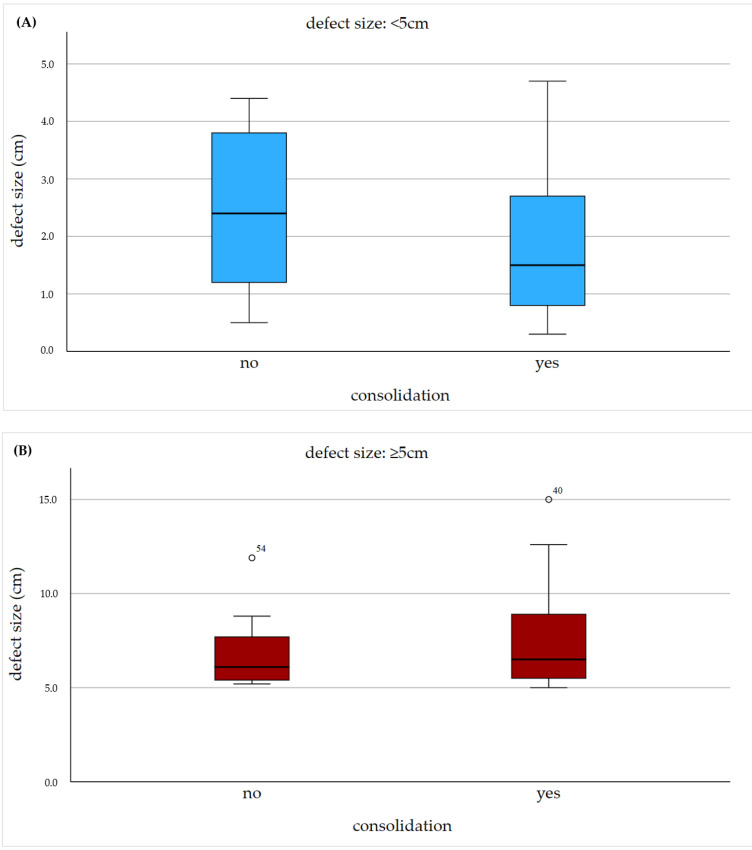
Radiological outcome in the context of the defect size. Visualisation of the defect size of the nonunions stratified based on consolidation after treatment is shown using box plots for the control group (**A**) and the study group (**B**).

The mean defect size was 7.13 cm (min.: 5 cm; max.: 15 cm; SD: 2.29) in the study and 2.09 cm (min.: 0.3 cm; max.: 4.7 cm; SD: 1.34) in the control group. The study group showed a consolidation rate of 53.8% whereas the control group showed a rate of 66.7% (Figure 4 and Figure 5). The chi-square test showed no significant difference regarding the consolidation rate (*p* = 0.247). In the overall collective with a defect size ≥5 cm, consolidation was achieved in 59.2%, indicating that the matched collective had a slightly worse consolidation rate. The consolidation rate in the overall control group was also slightly better, with 71.2%. Furthermore, logistic regression showed no correlation between an increasing defect size and the consolidation rate (*p* = 0.399). Patient characteristics of nonresponder to nonunion therapy are shown in Table 2. 

However, a higher number of previous operations showed a significant negative influence on the consolidation rate of the entire cohort (B = −0.109, *p* = 0.028). Likewise, a significant negative influence was shown with an increasing number of revisions (B = −0.905, *p* < 0.001). In particular, the study group showed a significant negative influence with regards to the number of revisions and the consolidation rate (B = −2.453, *p* = 0.004). The control group showed a slightly smaller negative effect of an increasing number of revisions on consolidation (B = −0.559, *p* = 0.013). Accordingly, it is not surprising that a rising total number of operations was also associated with a negative impact on consolidation (B = −0.191, *p* = 0.002). 

**Figure 6:**
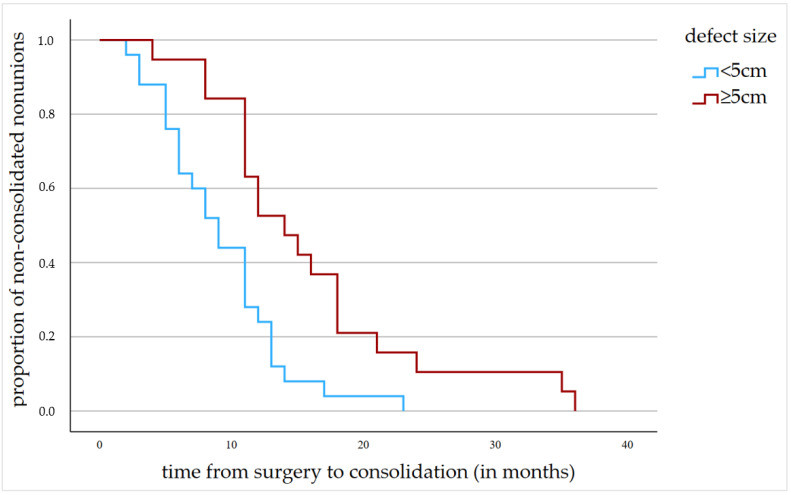
Kaplan–Meier curve showing the difference between the mean duration until consolidation was achieved in the study group and the control group.

Kaplan–Meier curve and log-rank test showed a significant difference regarding the mean duration until consolidation was achieved, with an average of 15.95 months (min.: 4 months; max.: 36 months; SD: 8.4) in the study and 9.24 months (min.: 2 months; max.: 23 months; SD: 4.8) in the control group (*p* = 0.001, Figure 6). The 95% confidence interval indicated a consolidation time between 7.3 and 11.1 months for the control and 12.2 and 19.7 months for the study group. Linear regression showed a significant increase in consolidation duration with increasing defect size (R^2^ = 0.121, *p* = 0.021). 

The mean duration until full weight bearing was achieved was 5.54 months (min.: 1 month; max.: 12 months; SD: 2.9) in the study and 4.86 months (min.: 1 month; max.: 11 months; SD: 2.2) in the control group. The t-test showed no significant difference between the average time to reach full weight bearing of the two groups (*p* = 0.267). Linear regression showed no significant correlation between an increasing defect size and time to full weight bearing (R^2^ = 0.005, *p* = 0.536). Furthermore, logistic regression showed no significant correlation between time until full weight bearing was achieved and the consolidation rate (B = −0.062, *p* = 0.498). Detailed logistic regression results can be found in Table 3.

### 3.2. Influence of Common Risk Factors on Nonunion Therapy Outcome

Previous smoking correlated negatively with radiological consolidation in all patients (B = −1.762, *p* = 0.043). Furthermore, the presence of diabetes mellitus, in a small numbers of cases, showed a subsignificant negative influence on the consolidation rate (B = −1.143, *p* = 0.091). The presence of an infection was associated with a subsignificant negative influence on consolidation (B = −0.847, *p* = 0.074). In the study group, a rising BMI correlated negatively with consolidation rate to a borderline significance (B = −0.150, *p* = 0.061). Neither age or sex, the exact anatomical localisation, the type of fracture (open vs. closed) or the type of nonunion (atrophic vs. hypertrophic) affected the outcome. In the study group, the use of NSAID showed a subsignificant negative correlation on consolidation (B = −1.435, *p* = 0.071). The use of steroids, drug or alcohol abuse and ASA score were observed to have no significant effect on consolidation. The detailed logistic regression results can be found in Table 3.

### 3.3. Influence of Therapy Modality

In all patients, the use of BMP2 correlated positively with the consolidation rate to a subsignificant level (B = 1.489, *p* = 0.066). The use of BMP7, on the other hand, had no effect on the outcome (B = −0.322, *p*= 0.488). The type of osteosynthesis (nail, plate or screws), the type of autologous bone graft (iliac crest, RIA, RIA + iliac crest) and the use or type of bone substitute (Vitoss^®^, Vitoss-BA, Bioglass^®^) showed no significant effect on the consolidation. Furthermore, the performance of a one-step or a two-step procedure did not affect the outcome (*p* = 0.596). 

## 4. Discussion

The aim of the current study was to determine the influence of defect size on the clinical and radiological outcomes of atrophic and hypertrophic nonunions treated with one-step or two-step procedures based on the diamond concept. We also tried to detect further risk factors for the consolidation and clinical outcome of large-sized bone defects. In addition, we analysed which complications are mainly to be expected with large bone defects in order to be able to take prophylactic countermeasures if necessary.

### 4.1. Impact of Defect Size on Radiological Outcome of Nonunion Therapy

In the absence of extensive literature on segmental bone defects, the effect of defect size on nonunion consolidation and clinical outcome remains unclear. Hsu et al. investigated the predicting factors for union and infection after applying the induced membrane technique (IMT) for segmental tibial defects [28]. According to their results, an initial infected nonunion and a defect length greater than 7 cm appeared to be risk factors for postoperative infection after Masquelet therapy. In addition, the presence of a postoperative infection was statistically associated with the development of a nonunion [28]. However, multiple logistic regression showed no direct association between the length of bone defect and the union rate [28]. Our results also showed no direct correlation between defect size and consolidation rate. Moreover, in our analysis, an infected nonunion was not considered a risk factor for a poorer radiologic outcome. Fung et al. presented a systematic review analysing the efficacy of the induced membrane technique and the relationship between patient factors and technique variations on the outcome [29]. Interestingly, they recorded increased odds for postoperative infection for each centimetre increase in defect size [29]. Furthermore, tibial fractures were associated with higher odds of infection when compared with femoral fractures and larger defect sizes were associated with higher odds of additional procedures [29]. Sun et al. postulated a higher risk of additional surgery for larger defect sizes in their meta-analysis [30], and El-Alfy et al. described a higher risk of additional procedures for bone defects larger than 7 cm [31]. In opposition to this, our study did not detect an increased risk of revision surgery with an increasing defect size, and the exact location did not affect the radiological outcome. However, there was a significant negative correlation between a higher number of previous surgeries, number of revisions or number of total surgeries of the affected limb and the consolidation rate. Karger et al. formed four different groups including a total of 84 patients with nonunions of different defect size (type I = less than 20 mm, type II = between 20 and 50 mm, type III = 50–100 mm, type IV = more than 100 mm) [24]. Analysis of the groups showed no differences in time to consolidation. Furthermore, similar consolidation rates between 88% and 95% were observed [24]. In agreement with this, we also found no significant differences in the consolidation rate. However, our data show that the time until consolidation increases significantly with increasing defect size. Another interesting finding was made by Aktuglu et al. in a narrative review of the Ilizarov bone treatment, describing a 3.7 times increase in the odds of re-fracture for tibial bone defects exceeding 8 cm [32]. Maceroli et al. investigated preoperative risk assessment scores for failure of bone grafting of tibia nonunions and segmental bone defects, including 203 patients who underwent surgery for nonunion or traumatic bone gaps [33]. According to the results, the size of the gap was a significant factor in the failure of surgery, with the probability of revision increasing by 6% for every 10 mm increase in the gap distance [33]. In comparison, we did not observe a higher risk of re-fracture or surgical failure with an increasing defect size.

### 4.2. Influence of Common Risk Factors on the Radiological Outcome

In agreement with our results, Tanner et al. showed that there was no significant effect of age on the consolidation rate [34]. Although smoking status was only of borderline significance, it is well known that active smoking has a negative impact on consolidation [8,35]. Furthermore, several studies detected a negative influence of the presence of diabetes on the outcome [1,34,35]. This was also seen in our analysis of the study group at a subsignificant level. In a large single-centre case–control study, Tanner et al. examined the impact of occult infection on the outcome of nonunion therapy. The presence of infection was associated with a prolonged time to consolidation and a slightly reduced consolidation rate, each at a subsignificant level [35]. Furthermore, an increased BMI showed a negative effect on consolidation [35]. Similar to this study, our study also showed a negative influence of occult infection in the control group, and in the study group, an increased BMI was associated with a negative influence on outcome, both with borderline significance. Hernandez et al. also reported an increased risk of fracture healing complications with NSAID use [1].

### 4.3. Limitations

This study has several limitations. Despite the large number of patients treated at our institution, there were only a small number of patients suffering a large-sized bone defect in the femur or tibia, resulting in a reduced number of patients eligible for matching. In addition, our strict matching criteria allowed us to find a suitable partner for few patients and we recognize that there might be a lack of power. Due to the fact that no other major studies on the influence of defect size on the treatment of nonunions are available, we believe that the results of the current study provide important insights for the treatment of such large-sized bone defects of the tibia or the femur. Surgeons should be aware of challenges and limitations of this type of treatment. Unfortunately, due to the fact that patients consult us for treatment from a large national and international catchment area, we also experienced a high rate of loss to follow-up. It could be suspected that selection bias exists due to the exclusion of patients who did not complete the follow-up. In addition, there is still the possibility of selection bias affecting the results of the current study, although we tried to reduce this risk by using blinded matchers. Due to the retrospective character of this study, we had to be confident that regaining full weight bearing was adequately documented.

## 5. Conclusions

Surprisingly, defect size does not seem to have a significant effect on the consolidation rate and should not be seen as a risk factor. However, for the treatment of large-sized nonunions, the follow-up period should be prolonged up to 24 months, due to the extended time until consolidation will be achieved. This period should also pass before a premature revision with new bone augmentation is performed. In addition, it should be kept in mind that as the number of previous surgeries and revisions increases, the prospects for consolidation decrease and a change in therapeutic approach may be required. In addition, common risk factors that lead to decreased bone healing such as the presence of diabetes mellitus or active smoking should be optimised. Further studies on the influence of defect size in radiological and clinical outcome of nonunion treatment need to be conducted.

## Figures and Tables

**Table 1:** Patient characteristics.

Characteristic	Group *	
	Bone Defect ≥5 cm (n = 39)	Bone Defect <5 cm (n = 39)
Age (years)	52.28 (±12)	52.33 (±12)
Defect size (cm)	7.13 (±2.3)	2.09 (±1.34)
ASA score	1.85 (±0.630)	2.10 (±0.502)
Sex		
Male	24 (61%)	24 (61%)
Female	15 (39%)	15 (39%)
Affected long bone and localisation		
Femur proximal	0 (0%)	0 (0%)
Femur diaphyseal	8 (20.5%)	8 (20.5%)
Femur distal	7 (17.9%)	7 (17.9%)
Tibia proximal	3 (7.7%)	3 (7.7%)
Tibia diaphyseal	7 (17.9%)	7 (17.9%)
Tibia distal	14 (35.9%)	14 (35.9%)
Nonunion treatment		
One-step	3 (7.7%)	3 (7.7%)
Two-step	36 (92.3%)	36 (92.3%)
Smoking status		
Yes	10 (25.6%)	10 (25.6%)
No	25 (64.1%)	25 (64.1%)
Previous	4 (10.3%)	4 (10.3%)
Infection		
Yes	19 (48.7%)	19 (48.7%)
No	20 (51.3%)	20 (51.3%)
Type of nonunion		
Atrophic	38 (97.4%)	38 (97.4%)
Hypertrophic	1 (2.6%)	1 (2.6%)
Type of fracture		
Closed	19 (48.7%)	27 (69.2%)
Open	20 (51.3%)	12 (30.8%)
Bone morphogenetic protein used		
rhBMP-2	7 (17.9%)	6 (15.4%)
rhBMP-7	17 (43.6%)	22 (56.4%)
None	15 (38.5%)	11 (28.2%)
Autologous bone graft used		
RIA (femur)	31 (79.5%)	27 (69.2%)
RIA (tibia)	2 (5.1%)	3 (7.7%)
RIA + iliac crest	2 (5.1%)	3 (7.7%)
Iliac crest	2 (5.1%)	4 (10.3%)
None	2 (5.1%)	2 (5.1%)
Bone substitute		
Vitoss	23 (59%)	28 (71.8%)
Vitoss-BA	4 (10.3%)	4 (10.3%)
Bioglass	6 (15.4%)	1 (2.6%)
None	6 (15.4%)	6 (15.4%)
Alcohol abuse		
Yes	7 (17.9%)	4 (10.3%)
No	32 (82.1%)	35 (89.7%)
Drug abuse		
Yes	2 (5.1%)	3 (7.7%)
No	37 (94.9%)	36 (92.3%)
Medication intake		
NSAID	10 (25.6%)	21 (53.8%)
NSAID + steroid	0 (0%)	1 (2.6%)
Steroid	1 (2.6%)	2 (5.1%)
None	28 (71.8%)	15 (38.5%)
Diabetes mellitus		
Yes	6 (15.4%)	5 (12.8%)
No	33 (84.6%)	34 (87.2%)
Method of osteosynthesis		
Nail	16 (41%)	17 (43.6%)
Plate	23 (59%)	21 (53.8%)
Screws	0 (0%)	1 (2.6%)
Consolidation		
Yes	21 (53.8%)	26 (66.7%)
No	18 (46.2%)	13 (33.3%)

* Age, sex, affected long bone and exact localisation, nonunion treatment, smoking status and infection were used as matching criteria.

**Table 2:** Patient characteristics of nonresponder to nonunion therapy.

Characteristic	Group	
	**Bone Defect ≥5 cm (n = 18)**	**Bone Defect <5 cm (n = 13)**
Age (years)	50.5 (±11.9)	51.46 (±11.7)
Defect size (cm)	6.73 (±1.8)	2.55 (±1.4)
Sex		
Male	11 (61.1%)	10 (76.9%)
Female	7 (38.9%)	3 (23.1%)
Affected long bone and localisation		
Thigh proximal	0 (0%)	0 (0%)
Thigh diaphyseal	4 (22.2%)	3 (23.1%)
Thigh distal	3 (16.7%)	1 (7.7%)
Tibia proximal	0 (0%)	0 (0%)
Tibia diaphyseal	4 (22.2%)	3 (23.1%)
Tibia distal	7 (38.9%)	6 (46.2%)
Nonunion treatment		
One-step	2 (11.1%)	1 (7.7%)
Two-step	16 (88.9%)	12 (92.3%)
Smoking status		
Yes	4 (22.2%)	4 (30.8%)
No	10 (55.6%)	7 (53.8%)
Previous	4 (22.2%)	2 (15.4%)
Infection		
Yes	10 (56.6%)	9 (69.2%)
No	8 (43.4%)	4 (30.8%)
Type of nonunion		
Atrophic	17 (94.4%)	13 (100%)
Hypertrophic	1 (5.6%)	0 (0%)
Type of fracture		
Closed	7 (38.9%)	9 (69.2%)
Open	11 (61.1%)	4 (30.8%)
Bone morphogenetic protein used		
rhBMP-2	2 (11.1%)	0 (0%)
rhBMP-7	8 (44.4%)	9 (69.2%)
None	8 (44.4%)	4 (30.8%)
Autologous bone graft used		
RIA (femur)	15 (83.3%)	10 (76.9%)
RIA (tibia)	2 (11.1%)	1 (7.7%)
RIA + iliac crest	1 (5.6%)	1 (7.7%)
Iliac crest	0 (0%)	1 (7.7%)
None	0 (0%)	0 (0%)
Bone substitute		
Vitoss	11 (61.1%)	10 (76.9%)
Vitoss-BA	2 (11.1%)	2 (15.4%)
Bioglass	2 (11.1%)	0 (0%)
None	3 (16.7%)	1 (7.7%)
Alcohol abuse		
Yes	3 (16.7%)	0 (0%)
No	15 (83.3%)	13 (100%)
Drug abuse		
Yes	1 (5.6%)	0 (0%)
No	17 (94.4%)	13 (100%)
Medication intake		
NSAID	7 (38.9%)	6 (46.2%)
Steroid	1 (5.6%)	1 (7.7%)
None	10 (55.6%)	6 (46.2%)
Diabetes mellitus		
Yes	4 (22.2%)	3 (23.1%)
No	14 (77.8%)	10 (76.9%)
Method of osteosynthesis		
Nail	6 (33.3%)	6 (46.2%)
Plate	12 (66.7%)	7 (53.8%)
Screws	0 (0%)	0 (0%)

**Table 3:** Results of logistic regression.

Variable	b	SD	*p*	OR	95% CI	
					LL	UL
Radiological and Clinical Nonunion Therapy Outcome						
Defect size (cm)	−0.062	0.074	0.399	0.940	0.813	1.086
Previous surgeries	−0.109	0.05	0.028 *	0.896	0.813	0.988
Revisions	−0.905	0.248	<0.001 *	0.404	0.249	0.658
Revisions (SG)	−2.721	0.901	0.003 *	0.66	0.11	0.385
Total surgeries	−0.191	0.061	0.002 *	0.826	0.734	0.930
Full weight bearing	−0.062	0.092	0.498	0.940	0.784	1.125
Influence of Common Risk Factors on Nonunion Therapy Outcome						
Previous smoking	−1.762	0.869	0.043 *	0.172	0.031	0.944
Active smoking	−0.258	0.545	0.636	0.773	0.265	2.250
Nonsmoking	0.258	0.545	0.636	1.249	0.444	3.769
Presence of diabetes	−1.143	0.677	0.091	0.319	0.85	1.201
Presence of infection	−0.847	0.474	0.074	0.429	0.169	1.084
BMI (SG)	−0.150	0.08	0.061	0.861	0.736	1.007
NSAID intake (SG)	−1.435	0.795	0.071	0.238	0.050	1.131
Influence of Therapy Modality						
Use of BMP2	1.489	0.808	0.066	4.431	0.909	21.597
Use of BMP7	−0.322	0.464	0.488	0.725	0.292	1.801
Type of procedure	0.452	0.852	0.596	1.571	0.296	8.339

* significant to α = 0.05, SG = study group analysis.

## Data Availability

Data was collected from our local non-union database of our Institution.
